# Protargol in Veterinary Surgery

**Published:** 1902-06

**Authors:** W. E. A. Wyman

**Affiliations:** Portland, Mich.


### PROTARGOL IN VETERINARY SURGERY.

By W. E. A. Wyman, M.D.V., V.S.,
PORTLAND, MICH.

(Concluded from page 273.)

15. *Punctured infected wound in front of patella, caused by pitchfork.* Two cases. Region of stifle enormously swollen; sanious discharge; no appetite; temperature, 103° F. and 104° F. respectively. The outer opening was enlarged, and the whole thing bubbled out with $H_2O_2$; a counter opening was made in the lower third of the internal face of the thigh; 10 per cent. protargol injections were ordered every two hours, with continuous hot water irrigations. Complete recovery in five and six days, respectively.

16. *Fistula on the internal face of the tarsal articulation following a kick.* The wound has discharged steadily for three weeks; 10 per cent. protargol injections three times daily. Complete recovery in fourteen days.

The writer is convinced that at best the injections were made once daily, as the whiskey injections which the owner habitually made and makes upon himself forbade punctuality on his part.

17. *Protargol* is of great value in infected wounds of the sensitive parts of the hoof; in fact, it is the only agent the writer so far employed successfully in the treatment of hoof wounds of a neurectomized hoof; that is, as far as such a hoof is capable of restoration. In deep, serious nail-pricks after curetting the nail tract, provided such treatment is warranted, the introduction of a cocoa butter protargol bougie gives good results, the pain disappears rapidly, and it is in these cases (92) that the writer imagined protargol to be possessed of anodyne qualities. Protargol has entirely replaced any other antiseptic as a dressing where the entire removal of the frog—

that is, the sensitive frog—is necessary, as in resection of the perforans tendon in necrosis from nail-pricks. In these cases, after resection of the perforans tendon, scraping of the os navicular, cartilaginous gliding surface, etc., the whole field of operation is finally thickly dusted with protargol, and sterilized oakum tampons placed over it, which again are held in place by bandages. The hoof thus dressed is enclosed in a leather boot. Since using protargol in these cases the dressing is changed for the first time on the twelfth day; of course, the animal's temperature is observed daily. Almost invariably the œdematous state of the subcutaneous tissue along the shin of the operated hoof previous to the operation will disappear entirely about the sixth day, while a healthy lot of granulations without any pus will greet the examining eye on the twelfth day. It is well to soak the hoof thoroughly with warm antiseptic solution the day previous to the first removal of the bandage, as the blood and protargol cause everything to stick tightly. That such an operation as resection of the tendon is done lege artis, and no jack-knife-style asepsis indulged in, need hardly be mentioned.

18. *Quittors.* As long as the necrotic tissue does not lie below the level of the coronary band, protargol injections will give quick and satisfactory results. Beyond that drainage is essential, and the quickest work is done by the knife. Certainly, this is a very general rule, and, therefore, not to be employed in every instance; nevertheless, it outlines the usefulness of protargol injections in quittors, and that is the only point under discussion at this moment. Generally speaking, all quittors which lame the animal but little ought not to be operated on, because protargol as given subject 5, injected about three times daily, will render good service. The time consumed by this injection treatment with the horse at work is from four to six weeks. In those cases where the pain and swelling are too great to permit of an immediate operation—that is, removal of the lateral cartilage—preparatory protargol injections soon will do away with the swelling and active pain, so much so that the owner often prefers the continuation of the protargol injections to extirpation of the lateral cartilage.

19. *Injuries to the coronary region,* especially when infection has taken place, often lead to serious consequences. Such injuries are frequent during cold and slippery weather, as the animals calk themselves with the sharp shoes, and, as a rule, infect the wound at the same time. The diffuse phlegmonous states and gangrenous processes spreading to the sensitive laminæ and leading to ungulation, cause death. A penetrating and strongly bactericidal agent is here desirable, and protargol is the remedy par excellence. After removal of the hair and all lacerated tissues the wound is thoroughly disinfected by a creolin, or, better yet, formalin bath ; next, the wound is covered with absorbent cotton, which is kept moist with the protargol preparation given subject 5. This dressing is permitted to remain for three or four days, unless the pain and swelling increase. In those cases with phlegmonous states of the subcutaneous connective tissue protargol or any other agent is likely to fail, as septicæmia or more likely pyæmia may bring on a fatal termination. The many observations made on injuries to the coronary region the past winter plainly show that protargol, without a question, is the safest, most penetrating bactericidal agent at present at the disposition of the surgeon in the treatment of injuries of that delicate part—the coronary region of the hoof.

In diseases of the eye protargol gave excellent results in all forms of purulent conjunctivitis, employed in 1 to 5 per cent. solutions, the treatment consisting in washing out the conjunctival sacs with a soft rubber bulb syringe once to three times daily. The œdematous state of the eyelids, as a rule, disappeared in two to six days, the purulent discharge rarely persisting longer than nine days. Ulcerations of the cornea as met with in distemper of the dog are readily handled by swabbing the part with a 10 per cent. protargol solution once daily for five days. Protargol in powder form has been used in a great many accidental wounds and in those made by the surgeon's knife. It readily adheres to the parts, forming a dry scurf, under which the process of healing, free from pus, takes place. The writer has used it instead of iodoform in numerous neurectomy wounds, healing by first intention being the rule, with better scar formation than any other antiseptic dressing, dry or moist, formalin being the only one which forms a close second.

Twice the writer has had the chance to prescribe protargol internally. The fact that it is not precipitated by dilute salt solutions, albumin, dilute hydrochloric acid, etc., puts its sphere of usefulness at once above nitrate of silver, and, outside of a slight constipating effect, nothing deleterious was observed. Of course, only two cases presented themselves, and while the action of the drug was all that could be wished for, the clinical material in this instance was too limited to permit of any positive deductions.

1. Large Newfoundland dog. Has been suffering with recurrent diarrhœas for six months. He was given a pill of 0.2 gramme protargol three times daily. In two days the stools became normal, and the animal was given 0.1 gramme once daily for one week. Results permanent so far (four months).

2. English setter. Hæmatemesis on and off, with colicky pains. Great loss of body weight since the past eight months. Was given 0.5 gramme in powder form. Vomiting of blood ceased in twenty minutes after the powder had been given. Next day the animal had quite a hemorrhage from the bowels, and received two powders of 0.5 gramme each, two hours apart. The next intestinal evacuation was free from blood. The animal after that was ordered 0.2 gramme daily for ten days. Remained well since (two months).

Summary: 1. The application of protargol, even to delicate parts, as the eye, seems to annoy the animal but momentarily. When applied to fresh wounds no inconvenience seems to be experienced by them at all.

2. It is a powerful and penetrating bactericidal agent.

3. It is a most active cicatrisant.

4. It is of special value in purulent infiltration of soft tissues.

5. In eczematous dermatitis of the lower phalanges it is the best remedial agent known to the writer, but has no special influence upon the course of dermatitis chronica verrucosa (greasy heels).

6. It gives superior results in the treatment of fistulous tracts, provided the general rules of surgery as to drainage, etc., are observed.

7. In otitis externa (canker of the ear) its action is astonishingly prompt and lasting.

8. Its peculiar properties render it continuously active, as it is not detrimentally influenced by the constituents of wound secretions.

9. Internally in gastro-intestinal ulcerations its action seems to be prompt and safe.

---

Etiology of Rinderpest. The microbe of rinderpest, which still remains to be cultivated, belongs, according to Nicolle and Adéle-Bey, to a group still invisible to the microscope. Two similar organisms are actually known, that of pleuropneumonia, discovered and cultivated by Nocard and Roux, and that of aphthous fever, discovered by Löffler, which until now has not been cultivated. The latter will pass through a Berkefeld filter while the former will not.

The germ of bovine pest, according to the authors who experimented with filtrations and injecting the filtrate, is intermediary in dimensions between the organisms of Nocard and that of Löffler.—*Comp. Rend. de l'Acad. de Paris.*

